# Effect of Tempering Temperature on Microstructure and Sulfide Stress Cracking of 125 Ksi Grade Casing Steel

**DOI:** 10.3390/ma15072589

**Published:** 2022-04-01

**Authors:** Ming Luo, Gao-Yang Zhou, Han Shen, Xin-Tian Wang, Mou-Cheng Li, Zhong-Hua Zhang, Guang-Hui Cao

**Affiliations:** 1 School of Materials Science and Engineering, Shanghai University, 99 Shangda Road, Shanghai 200444, China; luo_ming@baosteel.com (M.L.); zhougaoyang@shu.edu.cn (G.-Y.Z.); shen_han213@163.com (H.S.); xtwang7@163.com (X.-T.W.); 2 Baosteel Research Institute, Baoshan Iron & Steel Co., Ltd., Shanghai 201900, China; zhzhang@baosteel.com

**Keywords:** casing steel, 125 ksi, tempering temperature, mechanical property, sulfide stress cracking

## Abstract

The influence of tempering temperature on the microstructure of 0.5Cr0.4W steels was investigated by scanning electron microscope, and the roles of grain boundary character, dislocation, and Taylor factor in sulfide stress cracking (SSC) resistance were interpreted using the election backscattered diffraction technique. The 0.5Cr0.4W steels tempered at 690 °C, 700 °C, and 715 °C all showed tempered martensites. The specimen tempered at 715 °C exhibited a higher critical stress intensity factor (K_ISSC_) of 34.58 MPa·m^0.5^, but the yield strength of 800 MPa did not meet the criterion of 125 ksi (862 MPa) grade. When the specimen was tempered at 690 °C, the yield strength reached 960 MPa and the K_ISSC_ was only 21.36 MPa·m^0.5^, displaying poorer SSC resistance. The 0.5Cr0.4W steel tempered at 700 °C showed a good combination of yield strength (887 MPa) and SSC resistance (K_ISSC_: 31.16 MPa·m^0.5^). When increasing the tempering temperature, the local average misorientation and Taylor factor of the 0.5Cr0.4W steels were decreased. The reduced dislocation density, and greater number of grains amenable to slippage, produced less hydrogen transport and a lower crack sensitivity. The SSC resistance was, thus, increased, owing to the minor damage to hydrogen aggregation. Therefore, 700 °C is a suitable tempering temperature for 0.5Cr0.4W casing steel.

## 1. Introduction

In the 21st century, the new energy industry, e.g., clean and recyclable solar energy, shows great industrial potential and environmental benefits, but traditional fossil fuels are still an important part of the worldwide energy system. The petroleum industry is also a significant pillar industry in the international economy. At present, the oil and gas fields discovered in inland regions tend to have short supplies, after long-term exploitation, while the coastal and deep-sea areas abound in oil and gas. These areas are expected to be the future energy base [1,2]. However, the exploration and development of mineral products in these areas is subject to complex and harsh service circumstance, such as deep reservoirs, high temperature, high pressure, and strong corrosiveness [3,4,5]. Hydrogen sulfide corrosion is one of the main limitations to the exploitation of oil and gas fields. It will not only create localized and general corrosion on casing and tubing, but also causes brittle fracture accidents, such as sulfide stress cracking (SSC) and hydrogen-induced cracking (HIC) [6,7].

In the circumstances of enduring high internal and external pressure downhole, the electrochemical reaction between casing steel and its surrounding corrosive medium will slowly compound sulfide and a small amount of hydrogen. As there is H_2_S inside the tube, the hydrogen sulfide ion (HS^−^) and sulfide ion (S^2−^) will restrain the changing of hydrogen ion (H^+^) into molecular form. The free H on the surface of steel will permeate into the steel and accumulate at inclusions, precipitates, and dislocations, to form hydrogen pressure or blisters, decreasing the strength of the steel. Under external stress, cracks initiate and brittle fracture occurs, which is the basic reason for SSC [8]. Capelle et al. [9] evaluated the ability to absorb hydrogen of three API grade pipeline steels using a hydrogen electrochemical oxidation method. This indicated that all steels demonstrated a sensitivity to hydrogenating in deoxygenated, near-neutral pH NS4 solution under relatively ‘soft’ cathodic polarization. Recently, more and more emphasis has been placed on the isothermal aging used in pipeline steel. González-Arévalo et al. [10] reported that the changes in mechanical properties could be estimated using an artificial aging process, and that mechanical properties show a random behavior, represented by a generalized extreme value distribution, exhibiting considerable changes in variance and skewness at different aging times. The microstructure of steels determined the strength and hydrogen embrittlement resistance. From the perspective of chemical composition optimization and heat treatment improvement, tailoring the microstructure of steel is a feasible way to develop a high-performance oil casing tube. Previous studies reported that the quenched and tempered structure of microalloyed medium carbon steel has a favorable balance between strength and toughness [11,12]. Benefiting from the precipitation of fine precipitates dispersed in the matrix during the high temperature tempering, trapping and separating the hydrogen atoms in the steel enables the steel to have both high strength and excellent SSC resistance. It is promising to acquire high-strength and toughness oil tubular goods from through medium carbon Cr-Mo steel with adding quantities of W, V, and Ti for microalloying after proper heat treatments.

Oil tubular products have generally covered all the grades and specifications made by American Petroleum Institute (API); furthermore, a series including high strength and collapse, martensite corrosion, and H_2_S corrosion resistances, as well as high alloyed steels possessing an excellent performance with non-API, have also been developed [13,14,15]. With the continuous exploration and development of ultra-deep and medium-acid wells, API-5CT-C110 grade with specified minimum yield strength of 110 ksi (758 MPa) casing and tubing could not meet the requirement of high strength and sulfide resistance. It is known that, for pipeline steels, the resistance to hydrogen absorption decreases with the decreasing of steel strength (yield stress) [9]. In view of the oil tube service environment, it is imperative to develop and apply high grade tubing and casing to transport oil and natural gas from deep reservoirs. Our previous research optimized the composition of the non-API 125 ksi high grade (862 MPa) tubular products through particular Cr and W alloying, and the mechanical properties and SSC resistance have been greatly improved [16]. Synchronously, the related heat treatment needs to be further studied, while the influence of tempering temperature on the microstructure and properties also requires further investigation.

Based on non-API 125 ksi high grade 0.5Cr0.4W casing steel (composition shown in Table 1), in this study, three tempering temperatures were designed to tailor the microstructure, mechanical property, and SSC resistance. A standard test method set from the National Association of Corrosion Engineers (NACE), named the double cantilever beam (DCB) test, was utilized to evaluate SSC resistance quantitatively. The present paper describes scanning electron microscope (SEM) and election backscattered diffraction (EBSD) investigations, with particular an emphasis on the microstructure and crystallographic characteristics of grain boundaries, dislocations, and precipitates; and the effect of tempering temperature on the microstructure and SSC performance of 0.5Cr0.4W alloy was analyzed. Such a study is expected to be helpful in providing an effective strategy to achieve a high strength and SSC resistance in 125 ksi grade casing steel for industrial applications.

## 2. Materials and Methods

The tested steels produced by Baosteel Research Institute were melted in a vacuum induction furnace and cast into ingots; the chemical composition of the steel in the present study, after forging and rolling, is shown in Table 1.

**Figure 1:**
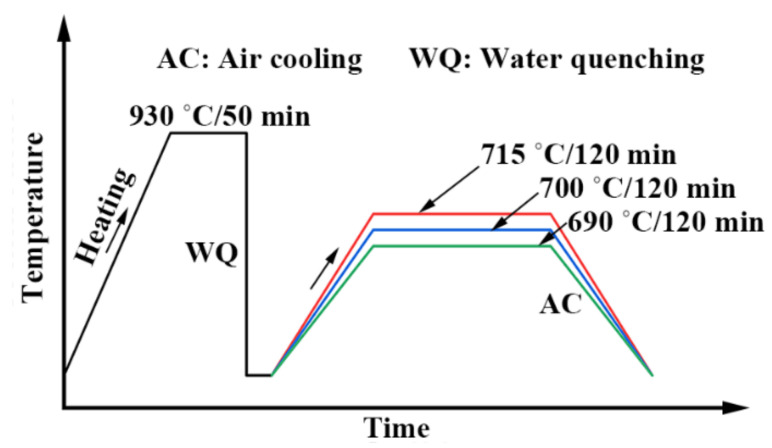
Schematic of the applied heat treatment routes of the 0.5Cr0.4W steels.

A schematic of the applied heat treatment routes of the 0.5Cr0.4W steels is presented in Figure 1. The tested steels were processed using a standard heat treatment method to obtain tempered martensite, which consisted of hot-rolling at 950 °C with air cooling, and quenching at 930 °C for 50 min with water cooling. Various tempering temperatures were selected: the tested steels were tempered at 690 °C, 700 °C, and 715 °C for 120 min with air cooling, respectively (the different spans were to maintain a similar gradient of ultimate tensile strength (Table 2) with decreasing temperature). The long-term tempering aimed to decrease the hardness and liberate the toughness, relying on dislocation annihilation and recovery, substructure evolution, and precipitation of carbides. The surface oxidation and decarburization formed in the hot processing on the rolled slabs were cut away.

The tensile test specimens were machined from rolled slabs in a direction parallel to the rolling direction, with the gage section of 40 mm in length and 6 mm in diameter. According to the ASTM Standard A370-2014, a tensile test was performed on an MTS C40 (MTS, Eden Prairie, MN, USA) electronic universal testing machine at a constant strain rate of 1 × 10^−3^ s^−1^ at ambient temperature. The yield strengths of all samples were identified using the stress corresponding to 0.7% offset strain. Hardness values were measured on a TH500 Rockwell hardness tester (Shanghai Optical Instrument Factory, Shanghai, China), with a load cell of 1500 N for 5 s, and each was tested 9 times and then averaged. The NACE-D method (DCB test) allows measuring the resistance of metallic materials to propagation of SSC, expressed in terms of a critical stress intensity factor (K_ISSC_), using a crack–arrest type of fracture mechanics test [17]. The DCB test does not depend on the uncertainty of pitting and/or crack initiation, because a crack is always initiated in a valid test. It gives a direct numerical rating of crack propagation resistance and does not rely on the evaluation of failure/no-failure results. Based on this method, the materials with higher K_ISSC_ values are deemed to possess superior SSC resistance performance. Our previous work described the detailed process of this experiment with casing and tubing steel [18]. Three groups of tempered steels were subjected to DCB tests, and three K_ISSC_ values were obtained for each group, with the average value being reported.

**Figure 2:**
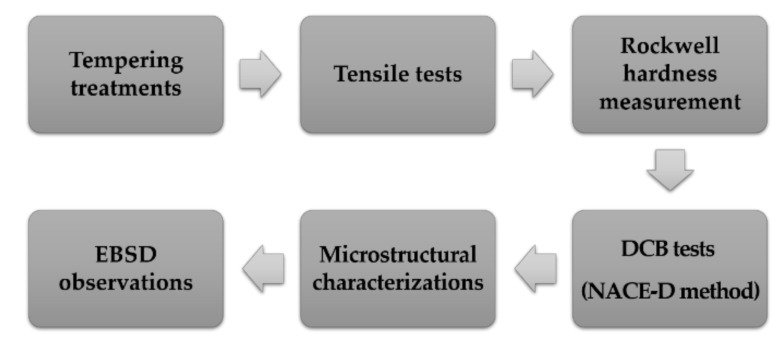
A flow chart of the experimental procedure.

The specimens selected for microstructural observation were cut from tempered steels by electric discharge machining in a size of 9 mm × 6 mm × 4 mm. The axes parallel and transverse to the rolling direction (radial direction) are defined as RD and TD, respectively, and the axis normal to the rolling direction is defined as ND. SEM observations were performed on a JSM-7001F (JEOL, Tokyo, Japan) with a field emission gun (FEG) operated at an acceleration voltage of 20 kV. The specimens for SEM analysis were ground with silicon carbide papers progressively up to 2000 grit and then polished and etched with a 4% nital solution. The grain orientation and grain boundary characteristics were investigated using a TESCAN MAIA3 FEG SEM (TESCAN, Brno, The Czech Republic) equipped with an EBSD detector and TSL OIM Analysis Software. The specimens at the RD–ND plane sections prepared for EBSD measurements. The specimens were polished with 0.05-µm silica gel solution in vibration polishing device for 6 h to remove surface stress. The sections of analysis were scanned with a step size of 50 nm at an accelerating voltage of 15 kV, and the sample was tilted at an angle of 70°. The complete experimental procedure is shown in Figure 2.

## 3. Results and Discussion

**Figure 3:**
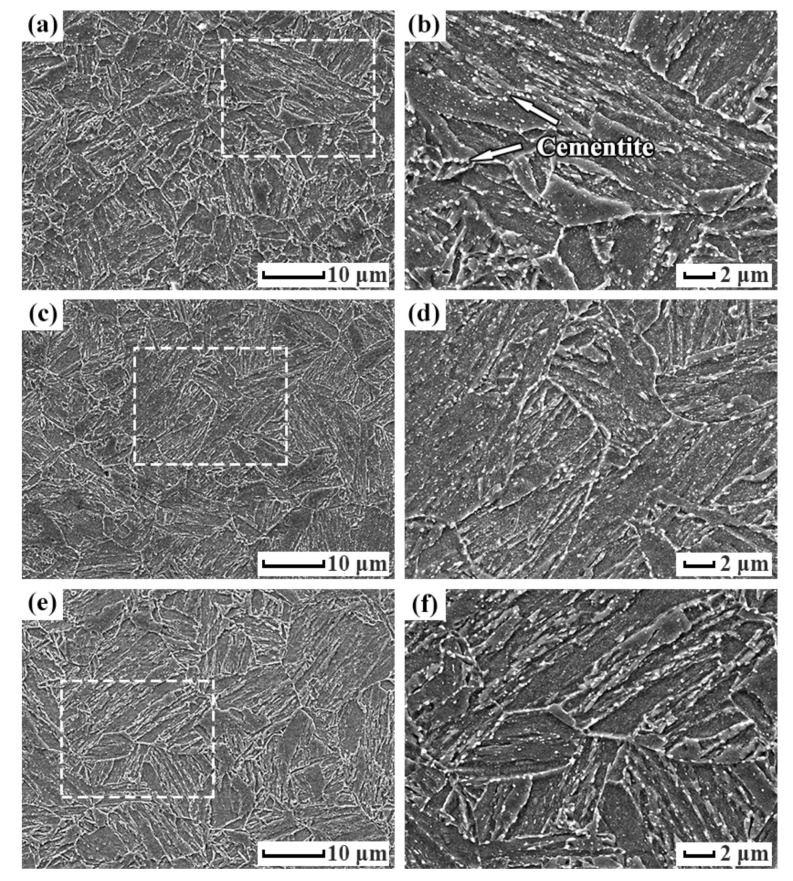
SEM micrographs of the 0.5Cr0.4W steels tempered at (**a**) 690 °C, (**c**) 700 °C, and (**e**) 715 °C, (**b**,**d**,**f**) are higher magnification SEM images of (**a**,**c**,**e**), respectively.

The microstructures of the tempered specimens at different temperatures characterized by SEM are shown in Figure 3. There are tempered martensites consisting of recrystallized ferrites and spheroidized cementites marked by arrows. Since the surface area of the sphere is the smallest, the carbides spontaneously spheroidized during the high temperature tempering process. The casing steel with this structure exhibited superior anti-hydrogen damage performance in an acid environment [19]. The sample tempered at 715 °C has more equiaxed ferrite grains (Figure 3e) of an average diameter in several micro-meters, superseding the original lath martensite morphology. The samples tempered at 690 °C and 700 °C still have some ferrites in their lath morphology, as shown in Figure 3a,c. Tempering temperature often has a major influence on various carbide precipitates in the thermomechanical treatment. High magnification SEM microscopy, as shown in Figure 3b,d,f, revealed that most white cementite precipitated in the interiors of the ferrites and along the grain boundaries are spherical, their sizes range from tens to hundreds of nanometers. The intergranular nucleation of the cementite is due to the grain boundary diffusion mechanism, which made cementite preferentially form at boundaries through diffusion phase transformation.

**Figure 4:**
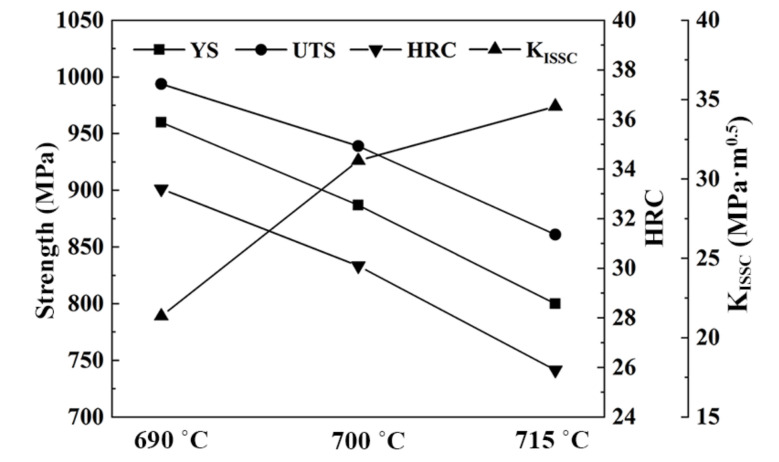
Mechanical properties and K_ISSC_ of the 0.5Cr0.4W steels tempered at different temperatures.

The yield strength (YS), ultimate tensile strength (UTS), hardness (HRC), and K_ISSC_ values of the tempered samples are presented in Table 2 and Figure 4. By analyzing the chart data, it is clear that the YS and UTS, and hardness decrease as the tempering temperature rises. Under 700 °C, tempered specimens fulfil the 125 ksi grade casing and tubing steel standards, which demands YS and UTS are higher than 862 MPa and 931 MPa, respectively. However, the specimen tempered at 715 °C could not meet the 125 ksi standard requirements.

However, the K_ISSC_ values had the opposite tendency to the above properties, indicating that the superior strength of steel was associated with the inferior SSC resistance. The K_ISSC_ value of the 690 °C-tempered sample was 21.36 MPa·m^0.5^, displaying poorer SSC resistance, while the K_ISSC_ for the sample tempered at 700 °C showed a reasonable SSC resistance of 31.16 MPa·m^0.5^. Although 715 °C-tempering enabled the K_ISSC_ value to reach 34.58 MPa·m^0.5^, the higher tempering temperature deteriorated the tensile strength excessively.

Previous investigations reported that the critical stress for SSC onset decreases with the increasing of yield strength [20,21]. Obviously, the SSC susceptibility of steel correlates with its strength or hardness, but steels with approximate strengths may exhibit different SSC performances [18]. Therefore, further investigation on the SSC behavior of casing steel in H_2_S media is necessary. The similar microstructures and characteristics of precipitates in the three tempered steels at different temperatures indicate that the crystallographic features of the specimens may have a significant effect on SSC performance.

**Figure 5:**
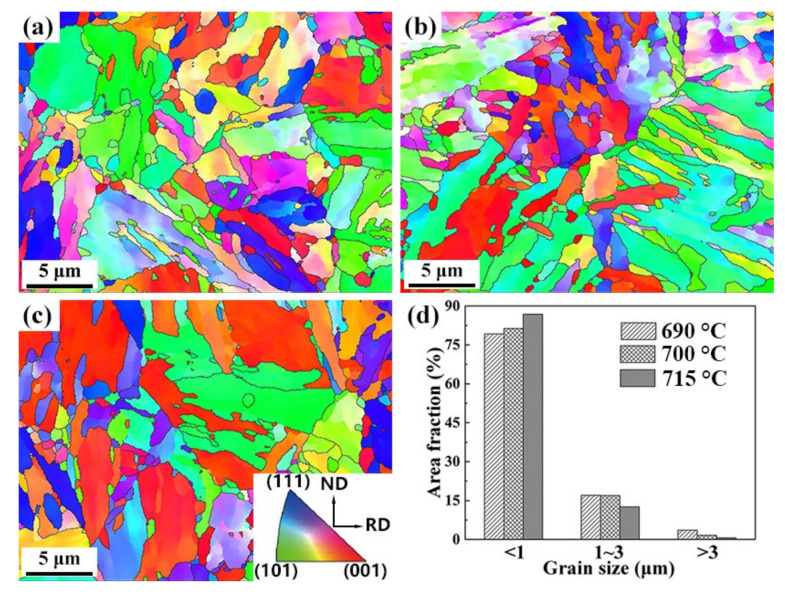
The grain orientation maps of the 0.5Cr0.4W steels tempered at (**a**) 690 °C, (**b**) 700 °C, (**c**) 715 °C, and (**d**) the histograms showing their grain size distributions.

Figure 5a–c shows the inverse pole figure (IPF) maps for the different temperature-tempered samples, and the number fraction of the grain size distribution obtained from EBSD data are shown in Figure 5d. IPF maps revealed that the orientations of grains in the samples were random after same the rolling and quenching process. There was no special texture observed in steels with different tempering temperatures. The statistical results in Figure 5d showed that with increasing tempering temperature, the proportion of grain size under 1 µm is dominant and gradually increases.

**Figure 6:**
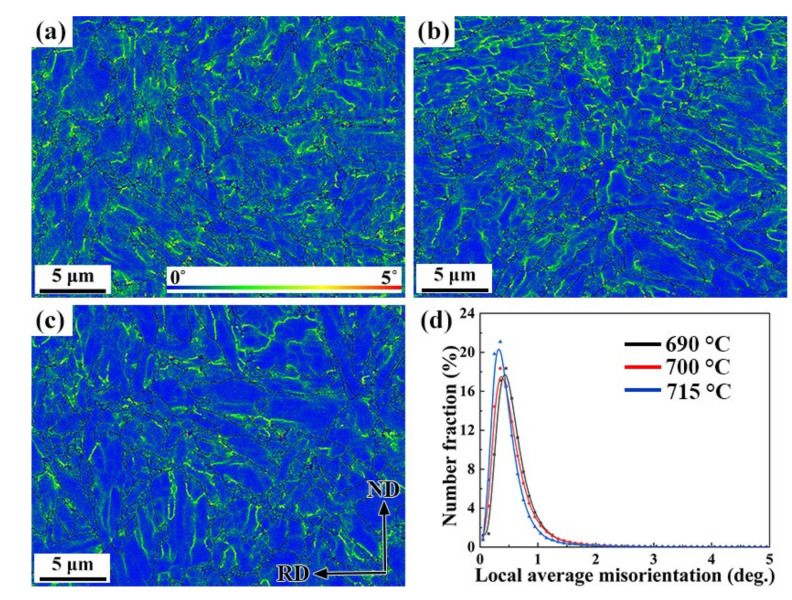
LAM maps of the 0.5Cr0.4Wsteels tempered at (**a**) 690 °C, (**b**) 700 °C, (**c**) 715 °C, and (**d**) LAM vs. number fraction.

The local average misorientations (LAM) for the three tempered samples are shown in Figure 6a–c. The LAM that represents the average misorientation among a given point and its nearest neighbors within the same grain, and, thus, associated with a misorientation under 5°, was utilized to characterize the local misorientation gradient [22]. In other words, LAM can be used to evaluate the magnitude of the local plastic strain, as well as the dislocation density [23]. In the color-coded LAM maps, the highest and lowest dislocation density areas are colored in red and blue, respectively. As shown in the maps, the deformation in steels is not uniform over the grain size scale and is obviously concentrated along the grain boundaries with high LAM values. The specimen tempered at 690 °C exhibited a higher LAM value than that of the other two specimens tempered at 700 °C and 715 °C. The LAM values vs. relative number of fractions are shown in Figure 6d, and the statistical curves comparing the LAM value and quantity ratio in the three tempered samples are also given. It is obvious that the LAM curves move to the left as the temperature rises, and the average LAM values of samples gradually decreased.

Dislocations in the martensitic substructure are the primary traps, and new grain boundaries resulting from the recovery and recrystallization of the ferrite matrix are the secondary traps for hydrogen. The average activation energy for hydrogen desorption from dislocations was 33.9 kJ/mol higher than the 25.2 kJ/mol from grain boundaries, which means that the dislocations trap hydrogen more easily than grain boundaries [24,25]. Therefore, the dislocation density exhibits a more significant impact on SSC resistance than grain boundaries, and a sample with a low dislocation density would be more immune to SSC [16].

Based on the above results, it is clear that the K_ISSC_ values of the tempered steels increase with the decrease of the average LAM values; therefore, as the dislocation density reduces, the SSC resistance of the steel increases. In accordance with the hydrogen enhanced localized plasticity (HELP) and hydrogen enhanced decohesion (HEDE) models [26,27,28,29,30], hydrogen can facilitate a dislocation motion and weaken the cohesion of Fe–Fe bonds, which promotes localized deformation and stress concentration. The absorption of hydrogen atoms by dislocations is insufficient to reach the threshold value for the formation of microcracks, while hydrogen can be transported to crack-sensitive regions, maintaining the dislocation motion [31]. Meanwhile precipitate in steel is an obstacle for dislocation movement, and incoherent interfaces between precipitates and the surroundings can act as trapping sites [32]. The trapped hydrogen atoms during the dislocation motion are released and located at the precipitate–matrix interfaces, due to the affinity between precipitates and hydrogen, which become the potential sites for crack initiation. Thus, it is believed that a higher dislocation density means a larger expressway for hydrogen to diffuse, and this may result in more microcracks being initiated in the steel.

**Figure 7:**
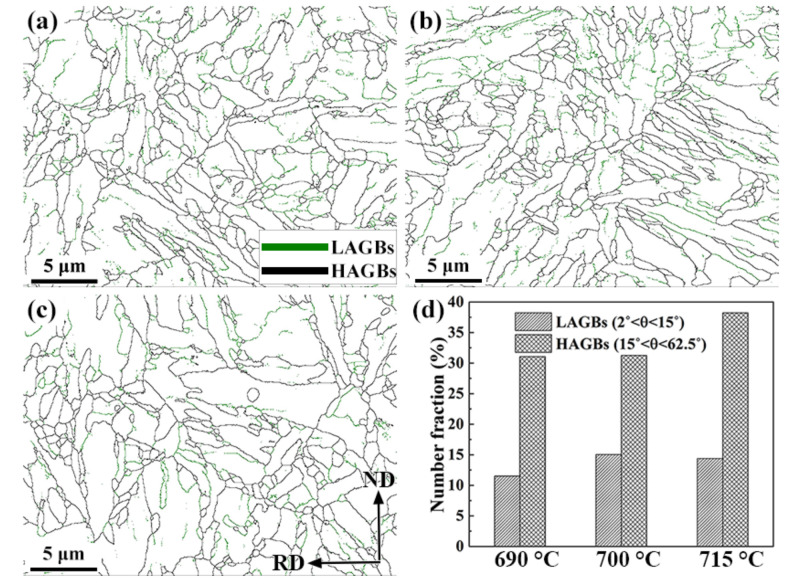
Grain boundary distributions of the 0.5Cr0.4W steels tempered at (**a**) 690 °C, (**b**) 700 °C, (**c**) 715 °C, and (**d**) LAGBs and HAGBs number fraction.

Figure 7a–c shows the distributions of low- and high-angle grain boundaries (LAGBs and HAGBs), marked in green and black lines with misorientation angles of 2° < θ < 15° and 15° < θ < 62.5°, respectively. The number fraction of these two types of grain boundaries is given in Figure 7d. Compared to the specimen tempered at 690 °C, the specimens tempered at 700 °C had more LAGBs and the proportion of HAGBs did not differ obviously. When the tempering temperature rose to 715 °C, the proportion of LAGBs decreased and HAGBs increased. It was previously reported that the total grain boundary area fraction in a unit volume increased after tempering at 700 °C, due to the changing of dislocations into polygonised boundaries [33]. Priority is given to the formation of LAGBs, which is a type of reversible hydrogen trap. In this regard, the hydrogen-induced cracks might not nucleate at LAGBs [34]. On the contrary, HAGBs have a higher activation energy, because of dislocation accumulation, and the absorption of dislocations into random boundaries leads to the development of HAGBs, which act as the preferential paths for crack propagation [35]. Recrystallization may take place over grains and boundaries during the tempering process, and the existence of second phase particles at the grain boundaries and their pinning effects will promote LAGBs transformation into HAGBs, where the smaller recrystallized grains will nucleate [36,37]. This is consistent with the statistical results in Figure 5d, whereby the proportion of grain size under 1 μm increased as the tempering temperature increased.

**Figure 8:**
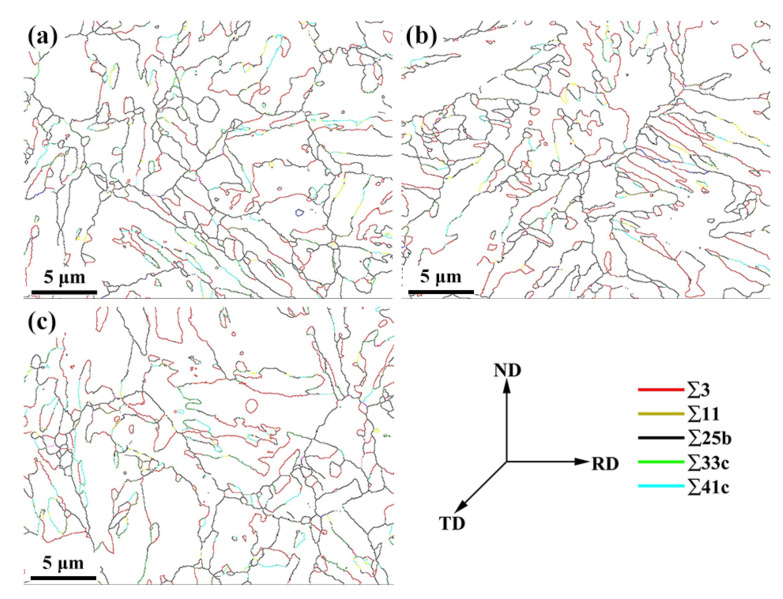
CSL maps of the 0.5Cr0.4W steels tempered at (**a**) 690 °C, (**b**) 700 °C, and (**c**) 715 °C.

**Figure 9:**
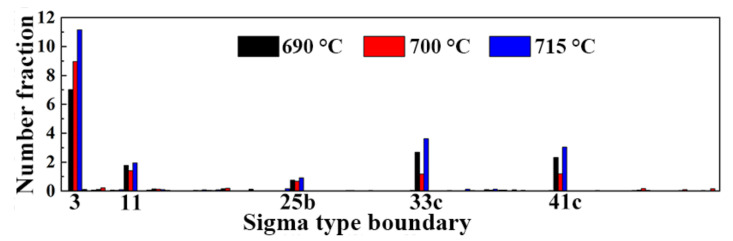
CSL map histograms of the 0.5Cr0.4W steels with different tempering temperatures.

The distribution of coincidence site lattice (CSL) boundaries in tempered samples are shown in Figure 8a–c, and the corresponding number fractions of Sigma-type grain boundaries are given in Figure 9. In this work, CSL boundaries were classified based on their Σ values, which is a measure of misorientation in coincidence site lattice theory [38]. As illustrated in Figure 9, Σ3, Σ11, Σ25b, Σ33c, and Σ41c are the primary CSL type grain boundaries in the three tempered specimens, and the relative fraction of the Σ3 boundaries is greater than that of other works. The W element in the tested steel suppresses the coarsening of the carbides and reduces the proportion of HAGBs and Σ3 boundaries, which change both the number of hydrogen traps and the capability for hydrogen trapping [16]. Among these CSL boundaries, we call particular attention to the Σ3 type, which are often thought to improve mechanical strength and resistance to stress corrosion cracking in metal alloys [39]. The Σ3 boundaries, as a kind of coherent twin boundary (CTB), which are considered to be inherently resistance to hydrogen embrittlement because of their high surface separation energy and low H solubility, play a dual role in H-assisted fracture. The Σ3 boundaries are weak against crack initiation and strong against crack propagation [40]. In other words, cracks may initiate at Σ3 CTBs, yet at the same time are unlikely to propagate along them. The fraction of the Σ3 boundaries increased with the tempering temperature in the test steels, and this tendency is consistent with the variation of K_ISSC_ values. Thus, we can assume that the effect of Σ3 boundaries in restraining crack propagation is greater than that the propensity for initiating crack.

**Figure 10:**
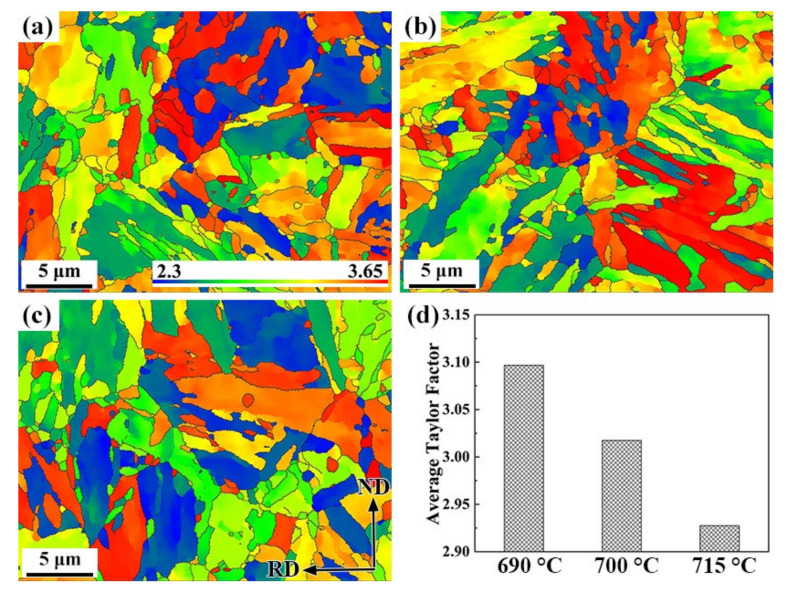
Taylor factor maps of the 0.5Cr0.4W steels tempered at (**a**) 690 °C, (**b**) 700 °C, (**c**) 715 °C, and (**d**) average Taylor factors.

The Taylor factor is always cited in the analysis of the plastic deformation of polycrystalline metals and implies the distribution of the grain orientations [41]. It is a powerful tool for predicting the difficulty of crystallographic slip of polycrystalline metals under various loading paths. In the present study, a uniaxial tension loading matrix in the direction of rolling was applied to the three specimens of primary and secondary slip systems for a bcc crystal lattice: {110} <111>, {112} <111> and {123} <111>. Taylor factor maps of the three specimens are shown in Figure 10a–c. The EBSD data of Taylor factors for the specimens processed by OIM software are inserted in the maps and illustrated with the column chart in Figure 10d. The average values of the Taylor factors begin to go down as the tempering temperature goes up. Under some given loadings, grains with lower Taylor factors inhibited crack propagation, for which slip was more likely to occur. A previous study showed that cracks arise mainly between grains with large Taylor factors and propagation ceases when the crack encounters a grain with a lower Taylor factor [42]. Furthermore, grains with high or similar Taylor factors are susceptible to trans-granular cracking, while grains with a mismatch in Taylor factors are prone to intergranular cracking [43]. It is well-accepted that grains are likely to crack when the grains cannot slip easily, causing stress concentration. The grains of the 690 °C-tempered steel with a higher Taylor factor were less likely to yield, leading to high dislocation density; therefore, hydrogen is transported to potential crack sites. As a result, sulfide stress cracking might occur, contributing to the external stress and H_2_S atmosphere. By contrast, the steel tempered at 715 °C with low Taylor factor exhibited a superior SSC resistance, but its mechanical properties were excessively deteriorated.

## 4. Conclusions

The microstructures of 125 ksi grade 0.5Cr0.4W casing steels tempered at different temperatures were studied by using SEM and EBSD, and their SSC susceptibilities were quantitatively evaluated using the NACE-D method. The influence of microstructural evolution and crystallographic features on mechanical property and SSC behavior were analyzed. The main conclusions are summarized as follows:

(1) The microstructures of the 0.5Cr0.4W casing steel tempered at 690 °C, 700 °C, and 715 °C were all tempered martensites, with good anti-SSC performance.
(2) With the price of deterioration in mechanical properties and hardness, the K_ISSC_ of steel tempered at 715 °C rose to 34.58 MPa·m^0.5^. Restricted by the strength limitation of 125 ksi grade casing steel, 700 °C is a more suitable tempering temperature option.
(3) The lower LAM exhibited a superior SSC performance; as the movable dislocation reduced under the external stress, less hydrogen was transported to the crack-sensitive zone weakening the detriments from local hydrogen concentration and SSC susceptibility.
(4) The average value of the Taylor factor went down as the tempering temperature rose, while for grain with a low Taylor factor, the slip is more likely to occur; effectively avoiding the stress concentration caused by entangled dislocation and inhibiting crack propagation.

## Figures and Tables

**Table 1:** Chemical composition of the 0.5Cr0.4W steel with errors (wt.%).

C	Si	Mn	Cr	Mo	W	Nb + V + Ti	Fe
0.26 ± 0.02	0.29 ± 0.03	0.52 ± 0.05	0.50 ± 0.04	0.80 ± 0.06	0.40 ± 0.04	(0.10–0.18) ± 0.03	Bal.

**Table 2:** The mechanical properties and K_ISSC_ values with standard deviations of the 0.5Cr0.4W steels tempered at different temperatures.

Tempering Temperature (°C)	Yield Strength (MPa)	Ultimate Tensile Strength (MPa)	Hardness (HRC)	K_ISSC_ (MPa·m^0.5^)
690	960/6.94	994/3.27	33.2/0.37	21.36/0.32
700	887/4.55	939/3.10	30.1/0.09	31.16/2.45
715	800/8.99	861/4.19	25.9/0.33	34.58/0.99

## Data Availability

Data are contained within the article.
